# Beyond the Textbook: An HBM and SCT Analysis of Adolescent Reproductive Health Education in Rural Bangladesh

**DOI:** 10.1002/puh2.70405

**Published:** 2026-10-04

**Authors:** Motasim Billah, Md. Forhad Uddin

**Affiliations:** ^1^ Department of Communication North Dakota State University Fargo North Dakota USA; ^2^ Department of Mass Communication and Journalism University of Barishal Barishal Bangladesh

**Keywords:** adolescent health, health communication, reproductive health education, school‐based health education, student engagement, teacher preparedness

## Abstract

The effective delivery of adolescent and reproductive health (ARH) education in rural Bangladeshi schools is influenced by factors extending beyond curriculum content, including teacher preparedness, classroom interactions, gender norms, and the broader social environment. This qualitative study examined how teachers and students experienced the delivery and discussion of ARH topics in rural secondary schools, drawing on the Health Belief Model (HBM) and Social Cognitive Theory (SCT) as interpretive frameworks. Data were collected through 18 in‐depth interviews with teachers and nine focus group discussions involving 54 students from six secondary schools across three rural districts. Thematic analysis identified substantial variation in teachers’ confidence and instructional practices, with training and administrative support associated with greater willingness to discuss sensitive topics and use participatory teaching approaches. Students’ engagement was shaped by gendered classroom dynamics, perceived approachability of teachers, and concerns about embarrassment. When students perceived communication with teachers as difficult, they frequently sought information from peers and online sources, despite uncertainty about the reliability of such information. Teachers also described adapting their instructional practices to navigate cultural sensitivities, whereas parental concerns, community expectations, curriculum limitations, and inconsistent school‐level support continued to constrain classroom discussion. Viewed through HBM and SCT, these findings illustrate how perceived barriers, self‐efficacy, social reinforcement, modeling, and institutional conditions interact in shaping ARH education. Strengthening teacher training, school leadership support, student‐centered communication, and community engagement may improve the delivery of ARH education in rural Bangladeshi schools.

## Graphical abstract

This qualitative study found that teacher preparedness, student engagement, and adolescent reproductive health (ARH) education in rural Bangladeshi schools are shaped by training opportunities, social norms, school support, and cultural barriers. Strengthening teacher training and institutional support may enhance the delivery and effectiveness of ARH education.

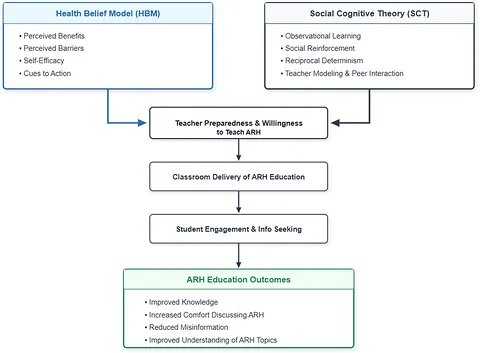

## 1 Introduction

Adolescent and reproductive health (ARH) is an important public health and human rights issue with implications for young people's physical, emotional, and social well‐being. Access to accurate and reliable ARH information remains challenging for many adolescents, particularly in low‐ and middle‐income countries (LMICs), where misinformation and limited access to supportive services can further complicate health‐related decision‐making. Adolescents aged 10–19 account for approximately 19% of Bangladesh's population [1], making their reproductive health needs an important public health concern. Persistent challenges related to child marriage, early pregnancy, and gender‐based violence further underscore the importance of accessible and culturally responsive ARH education. Bangladesh has taken steps to incorporate ARH into formal education, including the integration of ARH topics into secondary‐level physical education and health textbooks by the National Curriculum and Textbook Board (NCTB). However, the inclusion of ARH content in the curriculum does not necessarily ensure its effective delivery in classrooms. Teachers may experience discomfort, insufficient preparation, and community resistance, whereas students, particularly girls, may encounter social norms that discourage open discussion of reproductive health topics [2, 3].

These implementation challenges may be particularly salient in rural Bangladesh, where access to teacher training and educational resources can be limited and community expectations may shape how sensitive health topics are addressed in schools [4, 5]. Although comprehensive ARH education can support adolescents’ knowledge, attitudes, and health‐related decision‐making, its effectiveness depends in part on how teachers translate curricular content into classroom practice. Teachers, therefore, occupy an important position between formal curriculum requirements and students’ actual learning experiences. Yet, relatively limited attention has been given to how teachers in rural Bangladeshi schools perceive their preparedness to teach ARH and how they navigate sensitive topics in everyday classroom settings. Existing research in Bangladesh has examined different dimensions of ARH education, including teacher attitudes, parental perceptions, and curriculum content. However, these dimensions have often been considered separately, providing a limited understanding of how teachers’ and students’ experiences intersect within the classroom environment. Research simultaneously examining teacher preparedness, classroom practices, and student engagement in rural secondary schools remains limited. In addition, relatively little qualitative research has used the Health Belief Model (HBM) and Social Cognitive Theory (SCT) together to examine how individual, interpersonal, and contextual factors shape the delivery and experience of ARH education in Bangladesh. Addressing these gaps, the present study examines teacher preparedness and classroom practices alongside students’ experiences of discussing and seeking information about ARH within rural school settings.

This study draws on the HBM and SCT as complementary theoretical perspectives for understanding ARH education in rural school settings. HBM directs attention to perceived benefits, perceived barriers, cues to action, and self‐efficacy relevant to teachers’ willingness and confidence in addressing ARH topics. SCT extends this perspective by emphasizing observational learning, social reinforcement, reciprocal determinism, and the role of interpersonal interactions in shaping classroom behavior. Together, these frameworks provide a basis for examining how teacher preparedness, classroom practices, student engagement, and the surrounding social environment intersect in the delivery of ARH education. The study seeks to generate contextually grounded insights relevant to teacher training, curriculum implementation, and supportive school practices. Specifically, it addresses the following research questions (RQs):

1. How do teachers describe their preparedness and classroom practices for delivering ARH education?
2. How do students experience and engage in discussions of ARH topics with teachers and peers?
3. How do teachers navigate culturally sensitive ARH topics, and what barriers shape teachers’ and students’ experiences of ARH education?

## 2 Literature Review

### 2.1 Context and Importance of ARH in Bangladesh

ARH is widely recognized as an important component of public health and human development worldwide [6], particularly given the health and developmental needs of adolescents. It includes physical, mental, emotional, and social well‐being related to sexuality and reproduction rather than simply the absence of disease. The need to address ARH is particularly important in LMICs such as Bangladesh, where structural inequalities, cultural taboos, and limitations in policy and service provision contribute to persistent gaps in education and health services. In 2014, Bangladesh had approximately 47.6 million young people between the ages of 10 and 24, accounting for roughly 30% of the national population [7].

National surveys have consistently documented substantial challenges related to adolescent reproductive health, including child marriage, adolescent pregnancy, and reproductive tract infections [4, 5]. Early marriage is particularly consequential for reproductive health in Bangladesh because it can interrupt girls’ education and reduce their opportunities to access accurate sexual and reproductive health (SRH) information. In response to these concerns, the NCTB introduced ARH content into secondary‐level textbooks, primarily through the physical education and health textbook. The inclusion of ARH topics in the formal curriculum represented an important policy‐level recognition of the issue. However, the extent to which curricular inclusion translates into meaningful classroom teaching remains uncertain [2]. In practice, factors such as limited teacher preparation, social resistance, and inadequate resources continue to constrain effective implementation. Within the broader South Asian context, countries, such as Sri Lanka and Nepal, have adopted more structured approaches to school‐based SRH education [8, 9].

### 2.2 Teacher Preparedness and Classroom Delivery

Teachers play a central role in the effective implementation of ARH curricula because they are directly responsible for translating curriculum content into classroom instruction. Their knowledge, attitudes, and level of comfort can significantly influence how sensitive topics are discussed with students [3]. In Bangladesh, qualitative research has shown that many teachers feel uncomfortable discussing issues such as puberty, menstruation, and contraception, particularly in mixed‐gender classrooms. Such discomfort may result in teachers skipping certain topics or addressing them only briefly during classroom instruction [2, 10].

Evidence from other South Asian contexts points to similar challenges. Shahani et al. [9], for example, highlighted the importance of teacher attitudes in shaping SRH education in Sri Lanka. Although more than half of the surveyed teachers supported reproductive health education, a considerable proportion reported negative attitudes, with cultural norms and concerns about parental reactions creating barriers to discussing SRH content. Similar challenges have been documented in Bangladesh, where even trained teachers may avoid sections addressing physical changes during puberty because of anticipated discomfort or criticism [2]. At the same time, previous research suggests that appropriate training can help teachers overcome some of these barriers. Sadan and Jaicy [8] found that targeted training workshops improved teachers’ confidence, pedagogical skills, and willingness to discuss culturally sensitive topics, suggesting that teacher preparation can contribute to more effective ARH education. In Bangladesh, [4] evaluated a school‐based SRH program and found that adequately trained teachers covered the material more thoroughly and became more confident in responding to students’ questions both inside and outside the classroom. However, without consistent institutional and school‐level support, sustaining these improvements remained challenging.

### 2.3 Parental Attitudes and Community Norms

Parental support is an important factor in the success of school‐based ARH education. Rob et al. [10] found that Bangladeshi parents generally supported the inclusion of reproductive health education in school curricula. However, many parents had limited knowledge of reproductive and sexual health, which restricted their ability to discuss these issues with their children at home. This creates a situation in which parents support school‐based instruction but may be unable to reinforce what students learn outside the classroom. Community norms, often influenced by religious and cultural values, further shape both parental and teacher attitudes. Studies conducted by icddr,b found that stigma surrounding sexuality and reproductive health often discourages open communication between parents and adolescents [4, 5]. As a result, many adolescents may turn to peers or informal sources for information, increasing the possibility of receiving inaccurate information. These findings emphasize the importance of providing accurate and age‐appropriate ARH information through trusted educational settings such as schools. In rural communities, where teachers’ actions may be closely observed, concerns about community reactions can also discourage open discussion of sensitive topics [2].

### 2.4 Curriculum Gaps and Policy Barriers

Although the inclusion of ARH content in NCTB textbooks represents an important policy development, several gaps still remain. Akhter et al. [2] noted that although age‐appropriate information on puberty and hygiene is included, topics, such as consent, gender‐based violence, and reproductive rights, receive limited attention. Moreover, some content continues to reflect traditional gender stereotypes, which may limit the curriculum's potential to promote gender equality. The lack of a comprehensive national policy on ARH education also contributes to inconsistencies in implementation across schools. Without clear guidance and sufficient support, teachers may differ considerably in how they interpret and deliver ARH content. Previous research indicates that curriculum reform alone may not be sufficient and needs to be supported by teacher training, community sensitization, and adequate educational resources to achieve meaningful implementation [4, 11].

### 2.5 Theoretical Framework: HBM and SCT

To understand the dynamics of ARH education in Bangladesh, this study draws on the HBM and SCT. The HBM suggests that health‐related behaviors are influenced by individuals’ perceptions of susceptibility to a particular condition, the severity of its consequences, the benefits of taking action, barriers to action, cues to action, and self‐efficacy [12]. In Bangladesh, [13] applied the HBM to examine parental acceptance of the HPV vaccine and found that multiple HBM domains, including perceived susceptibility, severity, benefits, and cues to action, were significantly associated with acceptance, with perceived benefits and cues to action emerging as particularly strong predictors.

SCT complements the HBM by emphasizing observational learning, social reinforcement, and reciprocal determinism [14]. In the context of school‐based ARH education, SCT highlights how teacher modeling and peer discussion can shape student engagement and learning. When teachers approach sensitive topics with confidence and openness, they not only provide information but may also strengthen students’ confidence to discuss these issues. Combining these two frameworks allows for a more nuanced analysis: the HBM addresses individual‐level perceptions, whereas SCT considers the social and environmental influences on behavior. This combined approach is particularly useful for culturally sensitive areas such as ARH education, where personal beliefs and community norms are closely interconnected. Although other theories, such as the Theory of Planned Behavior, have been used in health communication research, integrating the HBM and SCT provides a broader lens for examining both teachers’ individual‐level perceptions and the classroom‐level social interactions that influence student engagement. Thus, these frameworks directly inform the study's RQs and coding scheme, connecting theoretical constructs with the lived realities of ARH education in rural schools. The conceptual framework guiding the study and illustrating the integration of the HBM and SCT is presented in Figure 1. Although the HBM includes six core constructs, the present study focused analytically on four constructs that were most directly relevant to participants’ experiences of ARH education: perceived benefits, perceived barriers, cues to action, and self‐efficacy. Perceived susceptibility and perceived severity were not used as primary analytic constructs because the study focused on classroom delivery and engagement rather than participants’ perceptions of personal susceptibility to or severity of a specific health condition. Accordingly, Figure 1 presents the four HBM constructs operationalized in the analysis.

**FIGURE 1:**
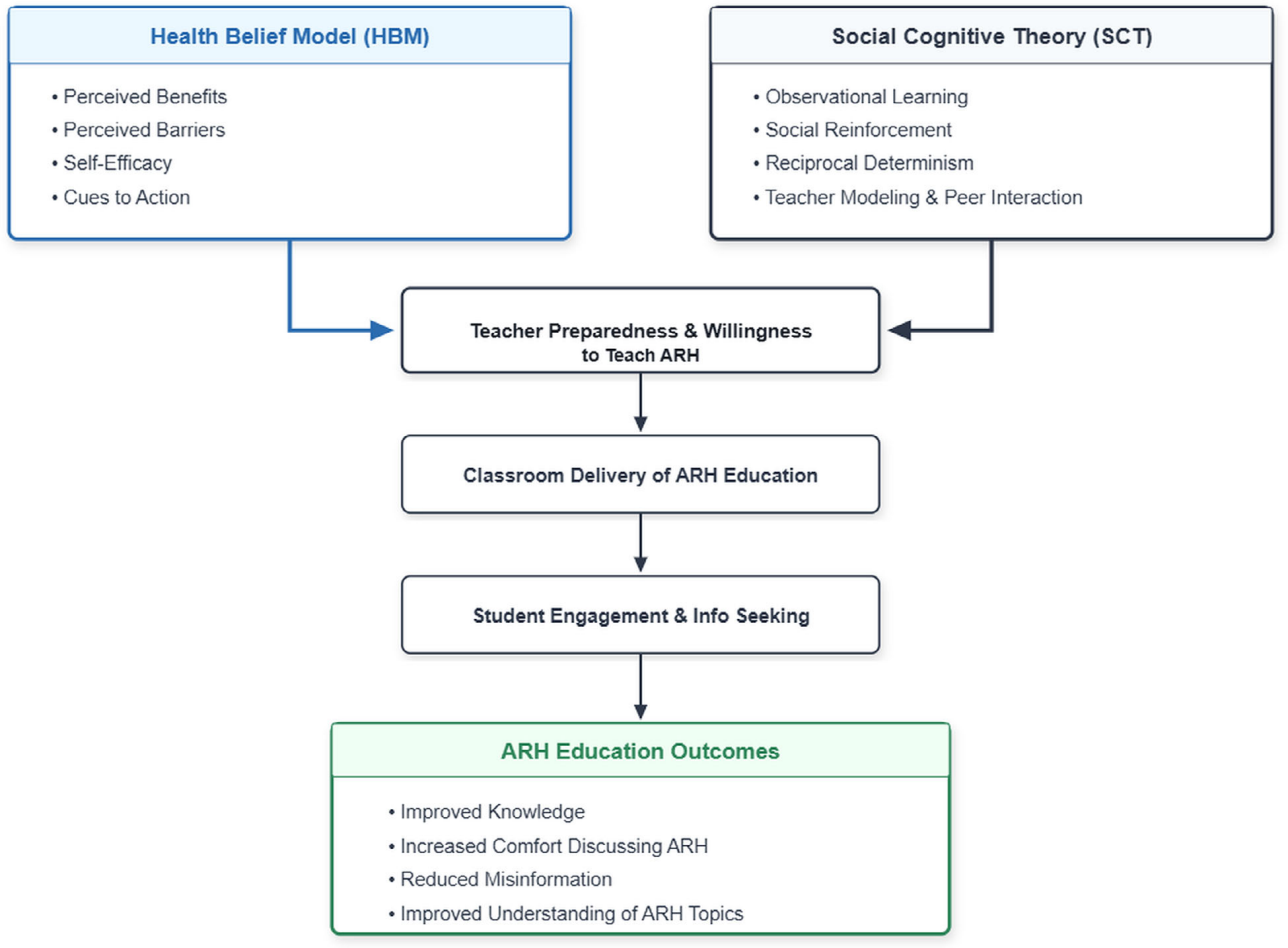
Conceptual framework illustrating how constructs from the Health Belief Model (HBM) and Social Cognitive Theory (SCT) informed the study's exploration of teacher preparedness, classroom interactions, and student engagement in adolescent and reproductive health education.

### 2.6 Research Gap

Although existing literature provides important insights into teacher attitudes [2], parental perspectives [10], and curricular limitations [2, 15], these factors have often been examined separately rather than holistically within actual classroom interactions. Moreover, research on ARH education in Bangladesh has largely focused on urban settings or non‐governmental organization (NGO)‐led pilot programs, leaving the experiences and dynamics of rural schools comparatively underexplored despite their heightened vulnerabilities. The use of theoretical frameworks such as the HBM and SCT in qualitative research on ARH education also remains limited, as much of the previous research has been primarily descriptive. This study addresses these gaps by examining how teacher readiness, student comfort, and societal taboos intersect to shape the delivery and reception of ARH education in rural Bangladeshi schools. By grounding the analysis in HBM and SCT, the study also seeks to provide both practical and theoretical insights into the factors shaping ARH education.

## 3 Methods

As the aim of this study is to capture the lived experiences of teachers and students who are engaged with ARH education in rural Bangladesh, the study employed a qualitative approach and interpretive design. Considering the RQs and purpose of this study, qualitative methods are well‐suited for exploring complex, context‐dependent phenomena [16]. The study was guided by the HBM and SCT, which informed both the development of data collection instruments and the coding framework used to address the study's RQs. In this study, a purposive sampling strategy was used to select participants who could provide diverse perspectives on ARH education in the context of rural areas. The sample comprised 18 teachers (10 female, eight male) and 54 students (29 female, 25 male) from Grades 6 to 10 across six secondary schools in three rural districts in Bangladesh. A summary of participant characteristics is presented in Table 1. Schools were selected to represent variation in type (public and private) and gender composition (coeducational and single‐gender). The primary language of instruction in the selected schools was Bangla (the native language).

**TABLE 1:** Characteristics of study participants.

Participant group	Number of participants	Gender distribution	Data collection method
Teachers	18	10 female, 8 male	18 in‐depth interviews (IDIs)
Students	54	29 female, 25 male	9 focus group discussions (FGDs)
**Total**	**72**	**39 female, 33 male**	**18 IDIs and 9 FGDs**

*Note:* Participants were recruited from six secondary schools across three rural districts in Bangladesh. Teachers participated in individual in‐depth interviews (IDIs), whereas students participated in focus group discussions (FGDs).

Teacher participants taught the physical education and health curriculum and had at least 1 year of experience delivering the NCTB curriculum. Student participants were selected to reflect variation in academic performance and socioeconomic backgrounds. Participant recruitment was conducted in coordination with the headteachers and relevant authorities of the participating schools. Teachers were invited directly to participate, whereas students were recruited through classroom announcements. As all student participants were minors enrolled in Grades 6–10, informed consent was obtained from their parents or legal guardians prior to participation. Students were informed in age‐appropriate language about the purpose and procedures of the study, the voluntary nature of participation, and their right to decline participation or withdraw at any stage. Administrative permission to conduct the study was obtained from the headteachers and relevant school authorities before data collection. Participation was voluntary, and no incentives were provided.

### 3.1 Data Collection

Data collection included 18 in‐depth interviews (IDIs) with teachers and nine focus group discussions (FGDs) with students. Four of the FGDs were gender‐segregated to encourage more open discussion. Each IDI lasted approximately 45–60 min, whereas each FGD lasted 60–90 min. All sessions were conducted in Bangla, took place in school classrooms, and were audio‐recorded with participant consent. Field notes were also used to document nonverbal cues, contextual factors, and researcher reflections. The interview guides addressed topics such as teacher preparedness, perceived barriers, strategies for discussing sensitive topics, and experiences with the ARH curriculum. The FGD guides explored students’ comfort levels, perceptions of classroom discussions, and suggestions for improving ARH education.

### 3.2 Data Analysis

Following the completion of the interviews and FGDs, all audio recordings were transcribed verbatim in Bangla to preserve the authenticity of participants’ expressions. The transcripts were then carefully translated into English for thematic analysis while maintaining the original context and meaning. The data were analyzed using the six‐phase thematic analysis framework outlined by [17]. The analysis began with repeated reading of the transcripts to develop familiarity with the data. Initial coding combined deductive and inductive approaches. Deductive codes were informed by the HBM constructs operationalized in this study, including perceived benefits, perceived barriers, cues to action, and self‐efficacy, as well as relevant SCT concepts such as observational learning, social reinforcement, and reciprocal determinism. Inductive codes emerged directly from participants’ narratives. Following initial coding, preliminary themes were developed by grouping related codes. Each theme was then reviewed for internal coherence and relevance to the RQs. The themes were subsequently defined and named to capture their central meanings, and representative quotes were selected to illustrate the findings in the final reporting.

To enhance the credibility of the findings, the study employed several strategies commonly recommended in qualitative research [18]. Data triangulation was achieved by drawing on multiple sources, including interviews with teachers and FGDs with students, allowing the researcher to compare and cross‐check perspectives across participant groups. Member checking was conducted with a subset of participants who reviewed and confirmed that the interpretations reflected their intended meanings. In a few cases, participant clarifications helped refine the wording of particular themes. The author also maintained reflexivity throughout the research process by critically considering how their own background, assumptions, and academic training might influence data collection and interpretation. An audit trail of coding decisions and thematic revisions was maintained to increase transparency in the analytical process. Together, these strategies strengthened the trustworthiness of the study by supporting credibility, reflexivity, and confidence in the interpretations presented. Ethical guidelines related to privacy, confidentiality, and voluntary participation were carefully followed, and pseudonyms are used throughout the paper to protect participants’ identities.

## 4 Findings

The analysis of the interviews with 18 teachers (*N* = 18) and FGDs with 54 students (*N* = 54) reveals nuanced insights into the state of ARH education in rural Bangladeshi secondary schools. The results are organized by the study's three guiding RQs. Across all themes, both teachers and students shared candid accounts of their experiences, which are presented here alongside interpretive analysis.

RQ1: How do teachers describe their preparedness and classroom practices for delivering ARH education?

### 4.1 Theme 1: Uneven Development of Teacher Confidence and Self‐Efficacy

The study found that for many teachers, addressing ARH topics posed a personal and professional challenge, particularly during the early stages of their careers. Several participants acknowledged that they had previously avoided certain lessons, especially those related to puberty, menstruation, and contraception, because of their own discomfort and concerns about possible reactions from students or parents. Over time, however, greater familiarity with the content and, in some cases, targeted training appeared to strengthen their confidence in addressing these topics.

When I first started, I avoided the puberty chapter. It was too awkward in front of boys and girls together. But now, after some workshops, I can talk about it more openly — still, there are topics I handle very carefully. (Amena, Female teacher, rural coeducational school)

Amena's experience illustrates how repeated exposure to ARH content and participation in training opportunities gradually increased her confidence in addressing sensitive topics. Although some hesitation remained, her account suggests that professional development helped reduce discomfort and strengthened her willingness to engage students in discussions that she had previously avoided.

After attending training provided by a non‐governmental organization, I realized that these topics are an important part of students' health education and improved my own understanding as well. Now I feel much more confident discussing puberty and menstruation than I did when I first started teaching because I understand their importance during adolescence. (Rabeya, Female teacher, rural coeducational school)

Rabeya's account further highlights the role of training in enhancing teacher preparedness. Beyond increasing factual knowledge, the training appeared to shift her perception of ARH education from a sensitive obligation to an essential component of adolescent well‐being. This increased awareness contributed to greater confidence in discussing topics that are often considered culturally sensitive.

This pattern reflects the HBM's self‐efficacy construct: teachers who felt confident in their ability to deliver the material were more likely to address it effectively. However, this development was not consistent across teachers. Those with limited training or previous experience with ARH content often remained hesitant and relied heavily on the textbook, with little elaboration or interactive discussion. Although this strategy helped teachers avoid potential embarrassment, it also limited students’ opportunities to develop a deeper understanding.

I usually follow the textbook closely because I am not sure how to explain some of these topics in detail or how much information I should provide. I worry about saying something wrong, creating misunderstanding among students, or facing questions from their parents. (Kamal, Male teacher, single‐gender school)

In contrast, Kamal's experience demonstrates how limited training and uncertainty can restrict classroom engagement. His concerns about providing inaccurate information and managing parental reactions encouraged a cautious, textbook‐centered approach. Such hesitation reflects lower levels of self‐efficacy and illustrates how fear of criticism may discourage teachers from expanding discussions beyond the prescribed curriculum.

### 4.2 Theme 2: Training as a Driver of Interactive Teaching Practices

Training and institutional support are important for the effective delivery of ARH content in the classroom. Teachers who had participated in structured training programs, particularly those provided by NGOs or supported by the Ministry of Education (MOE), demonstrated greater confidence and used more engaging classroom approaches. These training opportunities provided teachers with not only relevant knowledge but also practical teaching strategies for addressing sensitive topics.

Before training, I just read from the book. Afterward, I started using diagrams and examples from daily life. Students respond much better now. (Malik, teacher, single‐gender school)

Malik's experience illustrates how training can improve teaching practices beyond basic content delivery. Learning practical instructional techniques increased his confidence in using visual materials and examples from everyday life, which helped make ARH lessons more engaging for students. This indicates that training can strengthen both teachers’ knowledge and their capacity to discuss sensitive topics effectively. In these situations, observational learning from trainers (SCT), along with demonstrations of practical classroom strategies, appeared to encourage teachers to adopt more interactive approaches. In contrast, teachers without training often relied on reading directly from the textbook, resulting in less classroom engagement.

### 4.3 Theme 3: Teachers’ Perceptions of Rural–Urban Differences in Training Access

Although the study included only rural schools, several participants compared their own access to professional development with what they perceived to be available to teachers in urban settings. Participants commonly expressed the view that urban teachers had greater access to workshops, training opportunities, and teaching resources. These accounts represent rural teachers’ perceptions rather than a direct rural–urban comparison. As one participant stated, “Urban teachers get more chances for workshops, while we are left behind” (Jamil, Male teacher, rural public school). These accounts suggest that geographic differences in access to professional development were a concern among participants; however, the present study cannot establish the existence or extent of such differences because urban schools were not included.

RQ2: How do students experience and engage in discussions of ARH topics with teachers and peers?

### 4.4 Theme 4: Gendered Patterns of Student Participation

Gender norms played an important role in shaping students’ willingness to engage with ARH topics both within and beyond the classroom. Female students generally felt more comfortable approaching female teachers, particularly in private settings, to discuss sensitive issues such as menstruation and hygiene. In mixed‐gender classrooms, the presence and possible reactions of male peers often discouraged female students from participating openly. As one female student explained:

In class, I stay quiet because the boys will laugh. After class, I talk to Madam — she understands. (Female student, Grade 9)

In contrast, male students appeared more willing to ask questions during classroom discussions. However, their participation sometimes involved humor or teasing, which could further discourage female students from speaking openly about ARH topics.

When we ask Sir questions from the book, but sometimes we joke in the class, maybe that's why the girls don't join in for open discussion about the adolescent and reproductive health content in the class. (Male student, Grade 10)

These patterns can be understood through SCT's concept of social reinforcement, as students’ participation was influenced by the reactions and behaviors of their peers. From an HBM perspective, the possibility of embarrassment functioned as a perceived barrier to participation, particularly among female students. Thus, students’ comfort with ARH discussions was shaped not only by their individual willingness to participate but also by the gendered social environment of the classroom.

### 4.5 Theme 5: Teacher Approachability and Student Information‐Seeking

The findings highlight the importance of teacher approachability in shaping students’ willingness to engage with ARH topics. Students reported feeling more comfortable asking questions when teachers responded without judgment, respected their privacy, and treated their concerns seriously. These behaviors appeared to create a sense of trust that made it easier for students to discuss sensitive issues. As one female student explained:

She never laughs at our questions, even if they are silly. That's why I can talk to her. (Female student, Grade 8)

From an HBM perspective, such supportive teacher behaviors can function as cues to action, encouraging students to seek accurate information when they have questions or concerns. In contrast, when teachers appeared uncomfortable or dismissive, students were less willing to raise sensitive topics and were more likely to seek information from peers or other potentially unreliable sources.

### 4.6 Theme 6: Turning to Peers and Online Sources for ARH Information

When students felt uncomfortable approaching teachers or did not view them as safe sources for discussing sensitive issues, they often sought reproductive health information from friends or online sources. Peer networks within and outside school provided an accessible and familiar space for discussion; however, students recognized that the information exchanged among friends was not always complete or accurate.

When we feel shy to ask teachers, we usually talk with our friends first about the topic. Sometimes they know the answer, but sometimes everyone is confused what would be better answer or right information. (Female student, Grade 9)

This account shows how friends can become an initial source of information when students are hesitant to approach teachers. Although peers provide a comfortable and supportive space for discussing sensitive topics, students also recognized that their friends may have limited knowledge, increasing the possibility of confusion or misinformation.

Many students search on internet. We get information quickly, but we do not always know whether it is correct or not because we have limited option how to identify or verify right information because of our knowledge. (Male student, Grade 10)

Online sources similarly provided students with convenient and relatively private access to reproductive health information. However, participants described difficulty assessing the reliability of online content, particularly when they lacked the background knowledge needed to distinguish credible information from misinformation. Together, these findings indicate that when trusted adult guidance is unavailable or difficult to access, students seek ARH information through alternative channels. Although peers and online resources can make information more accessible, they can also expose students to incomplete or inaccurate information. This finding further emphasizes the importance of supportive classroom environments in which students feel comfortable seeking reliable information directly from trained teachers.

RQ3: How do teachers navigate culturally sensitive ARH topics, and what barriers shape teachers’ and students’ experiences of ARH education?

### 4.7 Theme 7: Adaptive Teaching Strategies for Sensitive ARH Topics

Several teachers described adapting their teaching practices to make ARH discussions less intimidating and more comfortable for students. They used relatively simple but creative approaches, including separate discussions for boys and girls, examples drawn from everyday life, role‐play, and group activities to encourage participation. These strategies helped create a more supportive classroom environment and provided students with opportunities to engage with topics that were often considered sensitive.

Sometimes I separate boys and girls for discussion because students feel more comfortable asking questions that way. But it is not possible all the time because of our limited time and resources. (Jaheda, Female teacher, rural government secondary school)

Jaheda's account demonstrates how teachers adjusted their instructional practices in response to students’ comfort levels. Separate discussions for boys and girls were viewed as one way to reduce embarrassment and make it easier for students to raise questions they might hesitate to ask in a mixed‐gender classroom. At the same time, her experience points to practical limitations, particularly time and resource constraints, that made it difficult to use this approach consistently.

I try to use examples from everyday life instead of directly using sensitive terms and I think this kind of example is more useful for them because these examples come from practical situations. It helps students participate without feeling embarrassed. (Halim, Male teacher, government secondary school)

Similarly, Halim described using familiar, everyday examples to make sensitive ARH topics easier for students to discuss. Connecting ARH concepts with situations students could recognize appeared to reduce discomfort and provide a less intimidating way to introduce topics that might otherwise be considered embarrassing or taboo. Overall, teachers showed flexibility and creativity in addressing culturally sensitive ARH topics. Their strategies focused not only on communicating information but also on creating classroom conditions in which students felt more comfortable participating. In this way, adapting instructional practices helped reduce communication barriers and created opportunities for more open discussion of ARH.

### 4.8 Theme 8: Institutional Constraints and Community Pressures

Despite their commitment to providing ARH education, teachers described facing substantial barriers arising from both the school environment and broader community norms. Participants explained that curriculum limitations, parental concerns, and community expectations often restricted how openly they could address ARH topics. Although many teachers recognized students’ need for more comprehensive information, they felt that institutional boundaries and social expectations limited the scope of classroom discussion.

Some parents think discussing reproductive health will encourage inappropriate behavior among students, and it is a very sensitive issue because we live in the same society. Because of that concern, teachers have to be very careful about what they talk about in their classrooms. (Kanij, Female teacher, Government secondary school)

Kanij's account demonstrates how community attitudes can influence teachers’ classroom practices. Teachers were conscious of how parents and other community members might respond to discussions of sensitive topics, which created pressure to approach ARH content cautiously. Consequently, concerns about possible criticism influenced both the scope and depth of classroom discussions.

Even when students want to know more, the curriculum does not cover everything, and sometimes we are afraid of criticism if we go beyond the textbook. There is a possibility that many parents might take it the wrong way if they hear about it because of our social taboos. (Jahid, Male teacher, Government secondary school)

Jahid's experience shows how limitations in the curriculum and concerns about criticism can operate together to restrict classroom discussion. Although teachers recognized that students sometimes wanted additional information, they often felt constrained by prescribed curriculum content and the possibility of negative reactions from parents or community members. These concerns made teachers hesitant to expand discussions beyond what was explicitly included in the textbook. Overall, the findings indicate that institutional constraints and community pressures continued to shape the delivery of ARH education in rural schools. Teachers had to balance their responsibility to provide students with useful health information against social expectations that discouraged open discussion of reproductive health. This tension often resulted in cautious treatment of sensitive topics and reduced opportunities for more comprehensive classroom learning.

### 4.9 Theme 9: Administrative Support and Teacher Confidence

School leadership emerged as an important factor shaping how openly teachers addressed ARH content in the classroom. Participants explained that support from head teachers and school administrators influenced their confidence and willingness to discuss sensitive topics. In schools where administrators actively supported ARH education, teachers felt more comfortable covering the curriculum comprehensively and responding to students’ questions. In contrast, uncertainty about administrative support often encouraged teachers to take a more cautious approach.

I don't know about other schools, but in our school our head teacher encourages us to teach the full curriculum and supports us if parents raise concerns about teaching details related to this issue. That makes it easier for us to discuss sensitive topics. (Halim, Male teacher, Government secondary school)

Halim's account demonstrates how supportive school leadership can facilitate ARH instruction. When teachers believed that administrators would stand behind their instructional decisions and help address concerns raised by parents or community members, they felt more confident discussing sensitive topics and engaging students in meaningful conversations.

In some situations, we avoid certain discussions because we are not sure whether the administration will support us if complaints arise from parents or other influential members of the community, as many people do not have a comprehensive understanding of this topic. (Kamal, Male teacher, single‐gender school)

In contrast, Kamal's experience illustrates how uncertainty about administrative backing can restrict ARH discussions. When teachers were unsure whether school leaders would support them in the face of complaints, they were more likely to avoid topics they considered potentially controversial, even when they believed such information would be useful for students. Overall, the findings indicate that administrative support can shape teachers’ confidence in delivering ARH education. Supportive school leaders can reduce concerns about possible community resistance and provide teachers with greater confidence to discuss sensitive topics more openly. Conversely, limited or uncertain administrative backing may increase teachers’ caution and restrict the depth of classroom discussion.

## 5 Discussion

This study examined teacher preparedness, student comfort, and barriers surrounding ARH education in rural Bangladesh through the combined theoretical lenses of the HBM and SCT. The findings suggest that teacher confidence is closely associated with training opportunities, whereas student participation is shaped by gender norms and trust in teachers. At the same time, the effectiveness of ARH education continues to be limited by structural, cultural, and institutional barriers. Taken together, these findings suggest that effective ARH education depends not only on curriculum content but also on the interaction among individual beliefs, social norms, and institutional support.

One of the key findings concerns differences in teachers’ confidence and preparedness. Many teachers described gradually moving from discomfort and avoidance toward greater confidence in discussing sensitive ARH topics, particularly after participating in training programs. This finding can be understood through the HBM concept of self‐efficacy, which suggests that individuals are more likely to engage in a behavior when they believe they have the knowledge and skills needed to perform it successfully [12]. Teachers who received training reported greater confidence in addressing topics such as puberty, menstruation, and reproductive health, whereas those without training often relied more heavily on textbook‐based instruction. This pattern is consistent with previous studies in Bangladesh and Sri Lanka showing that professional training can strengthen teacher confidence, pedagogical skills, and willingness to address sensitive reproductive health topics [4, 8, 9]. These findings therefore highlight the importance of regular ARH‐specific teacher training as part of effective curriculum implementation.

Participants also perceived differences between rural and urban settings in access to professional development opportunities and educational resources. Several rural teachers believed that their urban counterparts had more frequent access to workshops, training opportunities, and institutional support. Because the present study included only rural schools, these accounts should be interpreted as participants’ perceptions rather than evidence of an observed rural–urban disparity. Previous research in Bangladesh has documented differences in the implementation of educational initiatives across rural and urban settings [11]. However, direct comparative research involving both rural and urban schools is needed to determine the extent and nature of geographic differences in ARH teacher preparedness and training access.

Gender norms emerged as another powerful factor shaping student participation. Female students frequently reported discomfort discussing ARH topics in mixed‐gender classrooms and often preferred seeking information privately from female teachers. Male students, by contrast, were generally more vocal during classroom discussions, although humor and teasing sometimes contributed to female students’ reluctance to participate. These findings reflect SCT's concept of social reinforcement, whereby anticipated peer reactions influence individual behavior [14]. Within the HBM framework, fear of embarrassment functions as a perceived barrier that discourages participation. Similar gendered patterns have been documented in Bangladesh, where cultural expectations often limit girls’ willingness to openly discuss reproductive health issues [2, 4]. The findings suggest that creating safe and supportive classroom environments may be particularly important for encouraging female students’ engagement with ARH content.

Closely related to student participation was the importance of teacher approachability and trust. Students were more likely to seek information when teachers demonstrated empathy, maintained confidentiality, and responded to questions without judgment. Such behaviors served as cues to action within the HBM framework by encouraging students to actively seek information and support. From an SCT perspective, teachers who modeled openness and respectful communication established classroom norms that promoted dialog and participation. These findings are consistent with previous research showing that positive teacher‐student relationships play a crucial role in facilitating reproductive health education and reducing barriers to communication [2, 10].

Another important finding concerns the role of peer networks and online sources as alternative channels of information. When students perceived teachers as unapproachable or felt uncomfortable discussing sensitive issues in school, they frequently turned to friends or the internet for guidance. Although these sources provided accessibility and privacy, students acknowledged that the information obtained was not always accurate or reliable. This finding supports earlier studies in Bangladesh showing that adolescents often rely on peers and informal information networks when trusted adult guidance is unavailable [5, 10]. The reliance on alternative information sources highlights the potential consequences of communication barriers within schools and underscores the need to strengthen teacher‐student communication regarding ARH topics.

The findings also reveal that teachers actively employed a range of strategies to reduce discomfort and challenge social taboos surrounding ARH education. Gender‐segregated discussions, everyday examples, and participatory teaching methods were commonly used to encourage student engagement. These approaches reflect SCT's emphasis on observational learning and social modeling, as teachers attempted to create classroom environments in which students could discuss sensitive topics without fear of embarrassment. Such strategies also reduced perceived barriers to participation, a key concept within the HBM. The findings suggest that even within culturally conservative settings, teachers can successfully facilitate ARH discussions when they adopt contextually appropriate and student‐centered teaching approaches.

Despite these efforts, structural and cultural barriers remained significant obstacles to effective ARH education. Teachers frequently described concerns about parental reactions, community expectations, and curriculum limitations. Many felt constrained by social norms that discouraged open discussion of reproductive health issues, particularly in tightly connected rural communities. These findings support previous research documenting how cultural stigma and community resistance can limit both the content and delivery of reproductive health education in Bangladesh [2, 5, 19]. The persistence of these barriers suggests that improving ARH education requires not only teacher training but also broader community engagement and curriculum reform efforts.

Finally, the study identifies school leadership as a critical yet often overlooked factor influencing ARH education implementation. Teachers who received support from head teachers and administrators reported greater confidence in discussing sensitive topics and responding to student questions. In contrast, uncertainty regarding administrative support often led teachers to avoid potentially controversial discussions. These findings suggest that institutional support can reduce perceived risks associated with ARH instruction and strengthen teacher confidence. Although existing literature has primarily focused on curriculum content and teacher preparedness, the present findings indicate that leadership support may play an important role in shaping classroom practices. Future interventions should, therefore, consider school administrators as key stakeholders in promoting effective ARH education.

Overall, this study contributes to the existing literature by integrating teacher and student perspectives within a rural Bangladeshi context and by applying HBM and SCT to explain how individual beliefs, social interactions, and institutional conditions jointly influence ARH education. Unlike previous studies that focused primarily on curriculum content, parental attitudes, or teacher perspectives in isolation, the present findings demonstrate how these factors intersect within everyday classroom experiences. The study therefore provides both theoretical and practical insights that can inform future policy development, curriculum reform, teacher training initiatives, and school‐based interventions aimed at improving adolescent reproductive health education in Bangladesh.

### 5.1 Limitations

Despite its contributions, this study has several limitations. The study focused on three rural districts in Bangladesh, which may limit the transferability of the findings to other geographic and educational contexts, particularly urban schools. Although the qualitative design provided rich insights into participants’ experiences, it cannot determine the prevalence of these patterns across the broader education system. In addition, the presence of the researcher during interviews and FGDs may have introduced social desirability bias, particularly when participants discussed sensitive topics or evaluated their own practices. Future studies could employ mixed‐methods approaches and comparative rural–urban designs to further examine the factors influencing ARH education in Bangladesh.

## 6 Conclusion

This study explored how ARH education is experienced and delivered in rural Bangladeshi secondary schools from the perspectives of both teachers and students. Guided by the HBM and SCT, the findings suggest that teacher preparedness, student participation, and the effectiveness of ARH education are shaped by a complex interaction of individual, social, and institutional factors. Although many teachers demonstrated increasing confidence in addressing sensitive topics, significant challenges remain, including limited training opportunities, gendered communication barriers, reliance on informal information sources, cultural stigma, curriculum constraints, and inconsistent institutional support.

The findings have important implications for policy and practice. Strengthening ARH education in Bangladesh requires sustained investment in teacher training, curriculum improvement, and school‐level support systems. Schools should create safe and inclusive learning environments that encourage open discussion, reduce stigma, and promote trust between teachers and students. In addition, greater engagement with parents and local communities may help address cultural resistance and foster broader acceptance of ARH education. Overall, the study highlights that improving ARH education involves more than simply including content in the curriculum. Effective implementation requires trained teachers, supportive school leadership, and learning environments that enable adolescents to access accurate information and engage in meaningful discussion about their health and well‐being.

## Author Contributions

**Motasim Billah** contributed to the conceptualization, methodology, data collection, formal analysis, writing of the original draft, and manuscript revision. **Md. Forhad Uddin** contributed to the development of the theoretical framework, interpretation of findings, critical review and editing of the manuscript, and supervision of the revision process. All authors reviewed and approved the final version of the manuscript.

## Funding

The authors have nothing to report.

## Ethics Statement

This study was conducted in accordance with accepted ethical principles for research involving human participants. Permission to conduct the study was obtained from the relevant school authorities and headteachers prior to data collection. Participation was voluntary. No incentives were provided, and confidentiality and anonymity were maintained throughout the study.

## Consent

Informed consent was obtained from all teacher participants and from the parents or legal guardians of all student participants, who were minors.

## Conflicts of Interest

The authors declare no conflicts of interest.

## Data Availability

The qualitative datasets generated and/or analyzed during the current study are not publicly available due to ethical and confidentiality restrictions involving participants’ privacy but may be available from the corresponding author upon reasonable request.
